# Circunferência da Cintura: Um Parâmetro Desfavorável para a Saúde Vascular

**DOI:** 10.36660/abc.20220508

**Published:** 2022-07-29

**Authors:** Erika Maria Gonçalves Campana, Andréa Araujo Brandão

**Affiliations:** 1 Hospital do Coração SAMCORDIS Niterói RJ Brasil Hospital do Coração SAMCORDIS, Niterói, RJ – Brasil; 2 Clínica Seacor Niterói RJ Brasil Clínica Seacor, Niterói, RJ – Brasil; 3 Universidade do Estado do Rio de Janeiro Faculdade de Ciências Médicas Rio de Janeiro RJ Brasil Faculdade de Ciências Médicas da Universidade do Estado do Rio de Janeiro, Rio de Janeiro, RJ – Brasil

**Keywords:** Doenças Cardiovasculares/complicações, Pressão Arterial, Aterosclerose, Rigidez Vascular, Dislipidemias, Circunferência da Cintura, Análise de Onda de Pulso, Avaliação de Resultados (Cuidados de Saúde)

A obesidade tem papel central nas doenças crônicas não transmissíveis, pela sua alta prevalência e forte relação com fatores de risco como hipertensão arterial (HA), dislipidemia, diabetes mellitus, e com morbidade e mortalidade. Em especial, a obesidade abdominal tem sido reconhecida como um fator de risco emergente para um status inflamatório e pro-trombótico que se associa a aumento da prevalência de HA e do risco de eventos cardiovasculares (CV). Esses dados são de importância fundamental em um cenário de prevalência crescente de obesidade em adultos bem como entre crianças e adolescentes.^1,2^ Resultados do Estudo do Rio de Janeiro^2^ mostraram associação desfavorável entre níveis elevados da pressão arterial (PA) e excesso de peso em adolescentes e maiores valores de PA e de variáveis antropométricas e metabólicas na fase adulta jovem.

Nos últimos anos, o entendimento do *continuum* CV tem evoluído e a doença CV tem sido vista no contexto do dano vascular. Nesse, a disfunção endotelial, que resulta na doença aterosclerótica e suas complicações, envolve a camada média das grandes artérias, acometida por um processo de envelhecimento acelerado do vaso, resultando em rigidez arterial mais precoce e arteriosclerose, o que contribui para a morbidade e mortalidade CV.^3–5^

A velocidade de onda de pulso (VOP) é o parâmetro não invasivo de rigidez arterial mais estudado e com aplicação clínica reconhecida. Metanálise^6^ demonstrou que, para cada aumento de 1m/s na VOP, havia um aumento de 14% nos eventos CV totais, de 15% na mortalidade CV e de 15% na mortalidade total. Assim, a rigidez arterial é um forte preditor de eventos CV e de mortalidade total, de forma que as principais diretrizes mundiais recomendam a avaliação da VOP para a estratificação de risco CV.^1,3,4^

O artigo em questão de Guimaraes et al.,^7^ mostrou a relação positiva entre a circunferência da cintura (CC) e a VOP e *augmentation index* (AIx). Os autores sugeriram que a CC, aferida de maneira simples e barata, pode estar relacionada ao dano vascular e assim ter papel na avaliação do risco CV e contribuir para o tratamento precoce e a prevenção de doença CV.

Estudos^8–13^ sobre a associação entre rigidez arterial, PA e variáveis antropométricas e metabólicas têm mostrado resultados variados; admite-se que a ação conjunta dos fatores de risco CV é o principal determinante para o dano vascular.^5^

Em idosos, um estudo^8^ relatou associações significativas entre VOP, idade, PA, CC, massa corporal gorda e leptina; entretanto em regressão logística, apenas a leptina alta e a adiponectina baixa foram preditores de rigidez arterial. Outro estudo^9^ demonstrou que quanto maior a massa muscular, menor a rigidez arterial em idosos longevos, sem relação estatística entre VOP e composição corporal. Por outro lado, outro estudo^10^ mostrou associação da VOP com CC, relação cintura-quadril e área de gordura visceral, mas não com índice de massa corporal (IMC); na análise multivariada, apenas a relação cintura-quadril e área de gordura visceral mantiveram relação com a VOP.

Em adultos jovens, estudo sueco^11^ não encontrou associações entre espessura da íntima média de carótida e a composição corporal. Porém, a distensibilidade arterial mostrou associações mais fortes com as medidas de composição corporal tanto em mulheres como nos homens. Análise do Estudo do Rio de Janeiro^12^ em população adulta jovem encontrou correlação significativa e positiva da VOP com PA, IMC, e lipoproteína de baixa densidade (LDL-colesterol) e negativa com lipoproteína de alta densidade (HDL-colesterol) e adiponectina. Entretanto, na análise de regressão múltipla, apenas o sexo masculino e a PA mantiveram correlação significativa com a VOP. Estudo^13^ em crianças obesas mostrou que a estrutura e elasticidade arterial são afetadas negativamente pelo excesso de peso e níveis de PA.

Portanto, o artigo em questão^7^ traz contribuição relevante para o entendimento dos determinantes do dano vascular e o papel dos fatores de risco tradicionais e emergentes, incluindo variáveis antropométricas simples como a CC. Os autores apresentam informações fundamentais para o potencial uso futuro dessa medida como alvo terapêutico e como biomarcador da melhora da estrutura da parede arterial e efetiva redução do risco CV.
