# Supplementary material for: Circunferência da Cintura: Um Parâmetro Desfavorável para a Saúde Vascular
Source: Arq Bras Cardiol. 2022 Jul 29;119(2):265–6. [Article in Portuguese] doi: 10.36660/abc.20220508 (PMC9363067; doi:10.36660/abc.20220508)
Supplement: Translation

# Translation: Waist Circumference: A Parameter of Vascular Health

**Authors:** Erika Maria Gonçalves Campana, Andréa Araujo Brandão

**Affiliations:** 1 Niterói RJ Brazil Hospital do Coração SAMCORDIS, Niterói, RJ – Brazil; 2 Niterói RJ Brazil Clínica Seacor, Niterói, RJ – Brazil; 3 Rio de Janeiro RJ Brazil Faculdade de Ciências Médicas da Universidade do Estado do Rio de Janeiro, Rio de Janeiro, RJ – Brazil

**Keywords:** Cardiovascular Diseases/complications, Blood Pressure, Atherosclerosis, Vascular Stiffness, Dyslipidemias, Waist Circumference, Pulse Wave Analysis, Outcome Assessment (Health Care)

Obesity plays a central role in chronic noncommunicable diseases due to its high prevalence and strong correlation with risk factors like hypertension, dyslipidemia, diabetes mellitus, and morbidity and mortality. In particular, abdominal obesity has been renowned as an emerging risk factor for an inflammatory and prothrombotic state, which is associated with an increased prevalence of hypertension and increased risk of cardiovascular (CV) events. These data are of utmost importance in the scenario of increasing prevalence of obesity not only in adults, but also among children and adolescents.^1,2^ Results of a study conducted in Rio de Janeiro^2^ showed an unfavorable relationship of elevated blood pressure (BP) and excess weight in adolescents with increased BP levels and anthropometric and metabolic variables in young adults.

Over the last years, the understanding of the CV continuum has grown, and CV disease has been seen in the context of vascular damage. In this sense, endothelial dysfunction, which results in atherosclerotic disease and its complications, involves the tunica media of large arteries, affected by an accelerated aging of the vessel, resulting in early arterial stiffness and arteriosclerosis, which in turn contributes to CV morbidity and mortality.^3–5^

Pulse wave velocity (PWV) is the most studied non-invasive parameter of arterial stiffness with renowned clinical application. A meta-analysis^6^ showed that, for every increase of 1m/s in PWV, there was an increase by 14% in overall cardiovascular events, 15% in CV mortality and 15% in overall mortality. Therefore, arterial stiffness is a strong predictor of CV events and overall mortality, and the main international guidelines have recommended the measurement of PWV for CV risk stratification.^1,3,4^

The article by Guimarães et al.^7^ showed a positive relationship of waist circumference (WC) and PWV with augmentation index (AIx). The authors suggested that WC, measured in a simple and cheap way, may be associated with vascular damage, and hence play a role in the assessment of CV risk and contribute to the early treatment and prevention of CV disease.

Studies^8–13^ on the association between arterial stiffness, BP and anthropometric and metabolic variables have yielded varied results; it is believed that the combined action of CV risk factors is the main determinant of vascular damage.^5^

In the elderly, a study^8^ reported significant associations between PWV, age, BP, WC, fat body mass, and leptin. However, the logistic regression revealed that only elevated leptin and low adiponectin were predictors of arterial stiffness. Another study^9^ demonstrated that the greater the muscle mass, the lower the arterial stiffness in long-lived adults, with no statistical relationship between PWV and body composition. On the other hand, another study^10^ showed an association of PWV with WC, waist-hip ratio, and visceral fat area, but not with body mass index (BMI); in the multivariate analysis, only waist-hip ratio and visceral fat area were associated with PWV.

In young adults, a Swedish study^11^ did not find an association between carotid intima-media thickness and body composition. However, arterial distensibility had the strongest associations with body composition measurements in both women and men. Analysis of the Rio de Janeiro study^12^ revealed a significant positive correlation of PWV with BP, BMI, and low-density lipoprotein (LDL)-cholesterol, and a negative correlation with high-density lipoprotein (LDL)-cholesterol and adiponectin in young adults. Nevertheless, in the multiple regression analysis, only male sex and BP had a significant correlation with PWV. A study^13^ with obese children showed that arterial structure and elasticity are negatively affected by excess weight and BP levels.

Therefore, the article under discussion^7^ makes important contributions to the understanding of determinants of vascular damage and the role of traditional and emerging risk factors, including simple anthropometric variables like WC. The authors present key information for the potential use of this measure as a therapeutic target and as a biomarker of improvement of the arterial wall structure and effective CV risk reduction.
